# Unraveling the Signaling Secretome of Platelet-Rich Plasma: Towards a Better Understanding of Its Therapeutic Potential in Knee Osteoarthritis

**DOI:** 10.3390/jcm11030473

**Published:** 2022-01-18

**Authors:** Cristina Del Amo, Arantza Perez-Valle, Leire Atilano, Isabel Andia

**Affiliations:** 1 Regenerative Therapies, Bioprinting Laboratory, Biocruces Bizkaia Health Research Institute, Cruces University Hospital, 48903 Barakaldo, Spain; cristina.delamomateos@osakidetza.eus (C.D.A.); arantzaperez6@gmail.com (A.P.-V.); leire.atilanosantos@osakidetza.eus (L.A.); 2 Radiology Service, Interventional Ultrasound Unit, Cruces University Hospital, 48903 Barakaldo, Spain

**Keywords:** platelet-rich plasma, growth factors, cytokines, inflammation, knee osteoarthritis, biological therapies

## Abstract

Platelets and their secretory products play an important role in determining the balance between tissue repair and tissue damage. To obtain novel insights into the molecular composition of platelet-rich plasma (PRP) and contextualize them in knee osteoarthritis (OA), two different plasma formulations, namely PRP and platelet-poor plasma (PPP), were prepared from six healthy donors following a biobank-automated protocol. Inter-donor differences were analyzed, and pools were created before performing multiplexing protein arrays. In addition, PRP and PPP were prepared from six patients following our in-house protocols. Supernatants from PRP and PPP were harvested one hour after calcium chloride activation. Multiplexing protein arrays were performed in parallel for all plasma formulations. Results were normalized to fold change in relation to PPP and examined using Ingenuity Pathway Analysis Software. Bioinformatic predictions showed that PRPs constitute a signaling system with interrelated networks of inflammatory and angiogenic proteins, including but not limited to interleukin-6 and -8 (IL-6, IL-8), insulin like growth factor 1 (IGF-1), transforming growth factor beta, (TGF-b), and vascular endothelial growth factor (VEGF) signaling, underlying biological actions. Predictions of canonical systems activated with PRP molecules include various inflammatory pathways, including high-mobility group box protein (HMGB1) and interleukin 17 (IL-17) signaling, neuroinflammation, and nuclear factor-kappa b (NF-κB) pathways. Eventually, according to these predictions and OA evolving knowledge, selected PRP formulations should be tailored to modulate different inflammatory phenotypes, i.e., meta-inflammation, inflame-aging or posttraumatic inflammatory osteoarthritis. However, further research to discriminate the peculiarities of autologous versus allogeneic formulations and their effects on the various OA inflammatory phenotypes is needed to foster PRPs.

## 1. Introduction

Osteoarthritis (OA) is a common incurable disease with pain, joint stiffness, and progressive impaired mobility as the predominant symptoms. The prevalence of OA has doubled when comparing the pre- and post-industrial periods, even after controlling for main risk factors, i.e., obesity and ageing [1]. OA constitutes a growing worldwide problem; the World Health Organization (WHO) has listed OA as the fastest increasing major health condition and ranked it second as a cause of disability [2].

During the next decade, the number of people aged 60 years or over globally is projected to increase by 38%, and 2021–2030 has been declared by United Nations General Assembly as “The Decade of Healthy Aging” [3]. OA is prevalent in late middle-agedness, and it is often associated with reduced patterns of physical activity hindering an active lifestyle and precluding the health benefits attributed to exercise [4]. In the current context of demographic changes and pandemia, the pace of baby boomers undergoing retirement has accelerated the need of OA effective treatments to avoid joint failure, pain, and disability, eventually ending in knee replacement [5]. The latter is predicted to rise 855% by 2012–2050 [6], with half of the recipients under 65 years. Before undergoing joint replacement, these patients spend an average of 13 years in palliative treatments [7]. 

During the past years, PRPs have been used as infiltrative therapy to treat knee OA. Yet, at present, PRPs are not deemed enough effective to be recommended in the guidelines for knee OA management, issued by international research societies [8]. Even though, recent meta-analyses reveal significant differences when PRPs is compared to the most common intraarticular treatments, i.e., corticosteroids and hyaluronic acid (HA) injections [9,10]. PRP, which is formed by a complex pool of cytokines, has introduced a conceptual therapeutic change from the single molecule/drug to the multimolecular approach involving an evolution in research methodology.

The molecular intricacies of PRP signaling in OA are poorly explored. Platelets are cytoplasmic fragments released from the megakaryocyte in the bone marrow to the blood stream, where they circulate for 8–10 days before being removed by macrophages in the spleen and liver. Platelets contain several granules, mainly alpha-granules, which constitute a reservoir of signaling proteins that are released upon platelet activation and fibrin formation [11]. Actually, although not being the most abundant, alpha-granule proteins are the main functional components of PRPs. They are core elements of PRP therapies because of their involvement in tissue repair and inflammation. Based on these assumptions, both product configurations, i.e., PRP and the supernatant released upon plasma coagulation, contain a large pool of signaling proteins, and both are used as infiltrative therapies in knee OA [12].

Accordingly, intraarticular injections are based on several hypotheses: first, anabolic actions of growth factors (GFs); second, immune modulation, and third, anti-inflammatory effects of chemokines and subsequent anti-catabolic effects. However, PRP intrinsic molecular complexity hinders full understanding of which of these hypotheses are relevant to their mechanism of action in OA.

Autologous PRPs are safe and can reduce pain and improve function in some OA patient subsets [13]. However, further understanding of PRP signaling in parallel with elucidation of OA phenotypes (relevant to biological therapies) is necessary to improve the efficacy of PRP treatments [14]. Although current criticisms emphasized the lack of standardization of PRP formulations, pure PRP (PRP without leukocytes) is the most common formulation used in OA. To leverage our insights into leuko-reduced PRP intricacies, we assessed an array of PRP core proteins and examined the molecular pathways, which are predicted to be associated with these molecules and are relevant to OA pathology.

## 2. Materials and Methods

**Figure 1:**
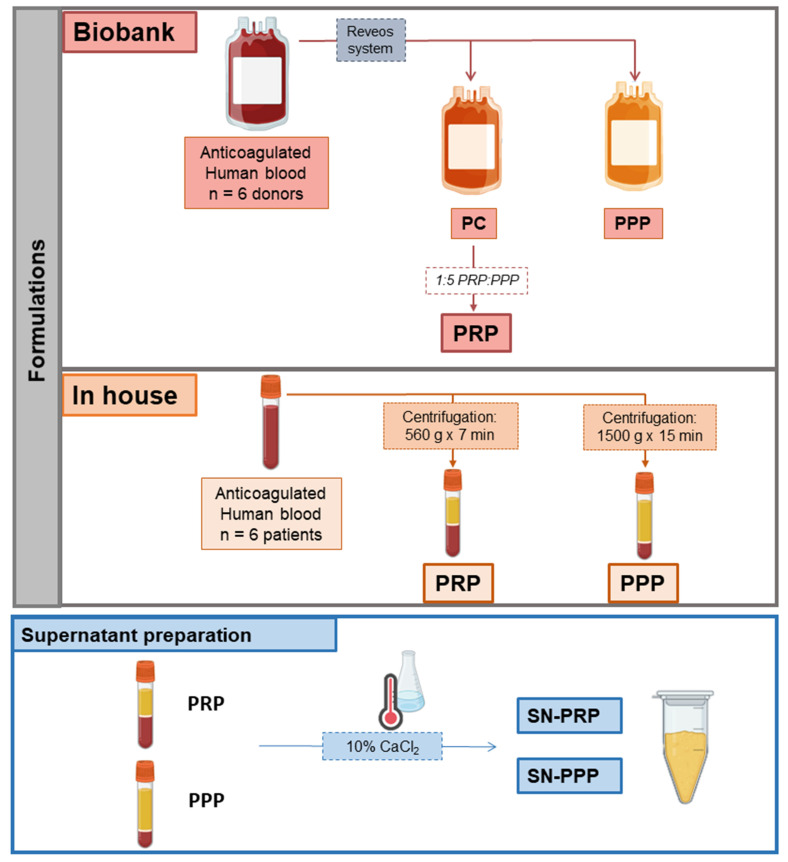
Diagram describing the procedure for platelet-derived products preparation. Biobank Platelet concentrate (PC) was diluted with platelet-poor plasma (PPP) to prepare platelet-rich plasma (PRP). In-house formulations were obtained by centrifugation of anticoagulated human blood. PRP and PPP supernatant (SN) were collected after CaCl_2_ addition and incubation at 37 °C for 1 h.

Two different protocols were used to elaborate two different plasma formulations, namely PRP and PPP. In addition, PRP and PPP supernatants, i.e., SN-PRP and SN-PPP, were prepared as described below (Figure 1).

### 2.1. Biobank PRP and PPP

Citrate phosphate dextrose (CPD) anticoagulated whole blood was obtained by apheresis from six healthy donors and processed by the Basque Biobank (Centro Vasco de Transfusión, Bizkaia, Spain; CEIC nº CES-BIOEF 201907). Leuko-reduced PRP and platelet-poor plasma (PPP) were prepared by means of REVEOS Automated blood processing system from Terumo Blood and Cell Technologies, BCT Inc. (Lakewood, CO, USA). PRP samples from blood bank were diluted 1:5 in PPP to homogenize the platelet range for quantitative analyses. Platelets were counted for each donor before further processing. According to Harrison et al., 2018, PRP classification system, biobank PRP product is L-PRP IIIC1, but after dilution, L-PRP IIIA1 (platelet concentration becomes category A, which is <900 × 10^3^ after dilution [15] and, according to Kon et al. [16], coding 29-00-10 and 24-00-10 after dilution with PPP.

### 2.2. In-House PRP and PPP

Anticoagulated (3.8% sodium citrate) peripheral blood was collected (Vacuette; Greiner BioOne, Kremsmünster, Austria) from six participants of the randomized clinical trial (NCT04231357, https://clinicaltrials.gov/ct2/show/results/NCT04231357?view=results, accessed 14 January 2022) approved by the Ethic Board (CEIm-E). All the volunteers positively responded to informed donation consent. Blood was centrifuged at 560× *g* for 7 min for PRP, and the plasma layer was collected avoiding aspirating the buffy coat. According to Harrison et al. 2018, PRP classification system, this product is PRP-IIA1 and, according to Kon [16], coding 24-00-10. Anticoagulated peripheral blood was centrifuged at 1500× *g* for 15 min for PPP preparation.

In both biobank and in-house PRPs, platelet activation was performed by repeated freeze and thawing cycles [17,18], then filtered through 40-µm filters, and stored at −80 °C before analyses.

### 2.3. Supernatants (Releasates) from PRP and PPP: SN-PRP and SN-PPP (in-House)

Supernatants were prepared by adding 10% CaCl_2_ final concentration 22.6 mM and subsequent incubation at 37 °C for one hour as described before (Releasate preparation, [19]); they were then centrifuged and the supernatant filtered through 40-µm filters and stored at −80 °C before analyses. In contrast to the products resulting from freeze-thawed procedures, which include fibrinogen, CaCl_2_ activation and subsequent centrifugation yields to the soluble protein fraction, the so-called releasate or supernatant.

### 2.4. Molecular Characterization

Total protein measurements were performed in biobank plasmas: PRP and PPP, in quadruplicates, using Pierce bicinchoninic acid (BCA) assay (Thermo Fisher Scientific, Waltham, MA, USA) following manufacturer’s instructions. In addition, total proteins were assessed in the supernatants SN-PRP and SN-PPP.

### 2.5. ELISAs

To quantify selected proteins relevant to inflammation and joint homeostasis and to determine inter-donor variability, ELISAs were performed in the individual biobank plasma samples according to manufacturer instructions. We studied the levels of human RANTES (CCL5) (900-K33; Peprotech Inc., Rocky Hill, NJ, USA), human VEGF (900-K10; Peprotech Inc., Rocky Hill, NJ, USA), human MCP-1 (CCL2) (900-K31, Peprotech Inc., Rocky Hill, NJ, USA), human PDGF-BB (900-K04, Peprotech Inc, Rocky Hill, NJ, USA), and human PF4 (CXCL4) (ab100628, Abcam, Cambridge, UK) in PRP and PPP.

### 2.6. Antibody-Based Protein Arrays

In order to assess core PRP proteins, we analyzed plasma formulations using a multiplexing antibody array platform, which detects human inflammatory cytokines and growth factors (126QAH-CAA-1000-1 Human Cytokine Array Q1000 Quantibody Human Cytokine Array 1000 Kit (RayBiotech Inc., Norcross, GA, USA)). The arrays were performed according to the manufacturer’s instructions, and the results were quantified against positive controls. To account for any biological variability, plasma samples from six donors were pooled. Array scanning was performed by the manufacturer service, and data were analyzed using Quantibody Q-Analyzer Software version 8.40.4 (Raybiotech, Peachtree Corners, GA, USA).

### 2.7. Bioinformatic Analyses

#### 2.7.1. Clustering Analysis

To detect the natural grouping of the samples studied in the arrays and visualize their differences according to the blood processing method, heat-map building was performed. For this, Perseus Software (1.5.1.6. version, Constellation Software Inc., Atlanta, GA, USA) was used. Before any data processing, data from the arrays were normalized to z-score to achieve Gaussian distribution.

#### 2.7.2. Ingenuity Pathway Analysis (IPA)

The proteins studied in the arrays were annotated and classified according to their GO functions. In order to analyze platelet secretome, fold changes were calculated, and each PRP product was normalized with the corresponding PPP product, i.e., biobank PRP fold-change in relation to biobank PPP dataset, in-house PRP fold-change in relation to in-house PPP, and SN-PRP fold-change in relation to SN-PPP. We evaluated the data set obtained from the multiplexing platform in the context of a large, structured collection of observations in various experimental settings with nearly 5 million findings manually curated from the biomedical literature or integrated from third-party databases, using the Ingenuity Pathway Analysis Software (IPA; Qiagen, Redwoood City, CA, USA). Signal pathway networks and canonical pathways were predicted using IPA algorithms.

To know the biological attributes associated with our datasets and not focused on statistical performance, we used a specific feature of IPA program; we added the datasets to “My pathways” and used the overlay tool to predict functionality for each dataset in the context of knee osteoarthritis.

## 3. Results

### 3.1. Plasma Formulations/Configurations

Biobank blood donors included four females and two males, with a median age of 52.5 years and range of 19–60. Platelet and leukocyte counts are shown in Table 1.

Blood donors for in house PRP included four female and two male patients with tendinopathy, randomized to the control group in the clinical trial (NCT) we conducted. The median age was 52 years (33–63). Platelet and leukocyte counts are shown in Table 2.

To examine inter-donor variations in the concentration of relevant plasma proteins, we performed individual measurements. The total protein content was similar in PRP and PPP samples (Table 3).

Paired comparisons between PRP and PPP (Mann–Whitney U) revealed significant higher concentrations in PDGF (*p* = 0.002), VEGF (*p* = 0.002), and RANTES (*p* = 0.002) in PRP compared to PPP. All measured proteins (except total protein) are contained in platelets’ alpha-granules; thus, there were significant correlations between them: PDGF and VEGF (*r* = 0.9232, *p* ≤ 0.001), PDGF and MCP-1 (*r* = 0.594, *p* = 0.042), and PDGF and RANTES (*r* = 0.783, *p* = 0.003) VEGF correlated positively with RANTES (*r* = 0.783, *p* = 0.003) as well as with MCP-1 (*r* = 0.706, *p* = 0.010).

As shown in Table 3, the concentrations of PDGF, VEGF, and RANTES are significantly higher in PRP than PPP, as they correlated positively with the number of platelets in the preparation. There are interdonor differences for several cytokines, and they are especially relevant for VEGF.

### 3.2. Multiplexing Protein Arrays

#### 3.2.1. Human Cytokine Array

Protein arrays showed similarities and differences between formulations based on the levels of specific growth factors and cytokines in the formulations. The most abundant proteins in PRP (range ng/mL) were IGFBP-1, -2, -3, -4, -6 and IGF-I, TGF-a, TGF-b1, TGF-b3, VEGF R2, and R3, VEGF-D, PDGF-AA, -BB, MCSF R, BDNF, NT-3, NT-4, FGF-4, FGF-7 and EGF-R; ICAM-1, IL-6sR, MIP-1d (CXCL9), RANTES (CCL5), TIMP-1 and TIMP-1, TNF-b, TNF RI, and TNF RII.

Fifty-two molecules were upregulated with relation to PPP (ratio > 2), including BDNF, EGF, FLT4, IFNG, and PDGF, all of which had the highest fold change (Table S1).

#### 3.2.2. Clustering Analyses

**Figure 2:**
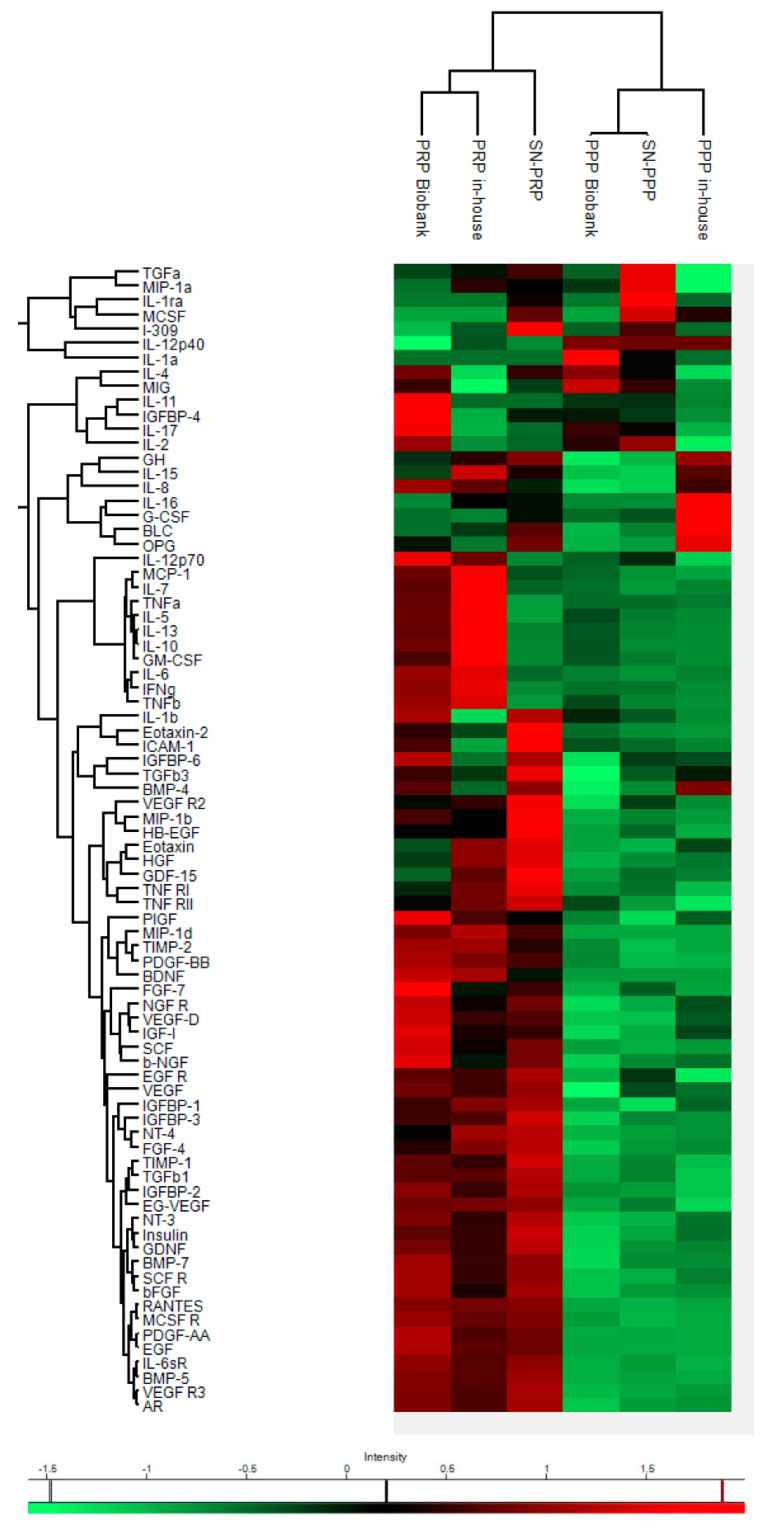
Heat-map shows differential expression of 80 proteins between biobank PRP, PPP, and in-house PRP and PPP as well as SN-PRP and SN-PPP. The color code indicates concentrations of GFs and cytokines expressed in pg/mL, ranging from red (high concentrations) to green (low concentrations).

Although hierarchical clustering showed different proteomic profiles of plasma formulations, heat map revealed two main clusters corresponding to plasma preparations with low (PPP Biobank, PPP in house, and SN-PPP) and high platelet content (PRP Biobank, PRP in house, and SN-PRP), as shown in Figure 2.

The main differences are attributed to the presence of platelets in the preparation (PRP vs. PPP). However, there were differences between protocols, i.e., “biobank” and “in-house” plasmas. These differences could be attributed not only to the preparation method but also possibly to donor differences (healthy vs. patients with tendinopathy).

#### 3.2.3. PRP Contains Signaling Systems (Networks) Involved in Immune Cell Interactions and Inflammation

**Figure 3:**
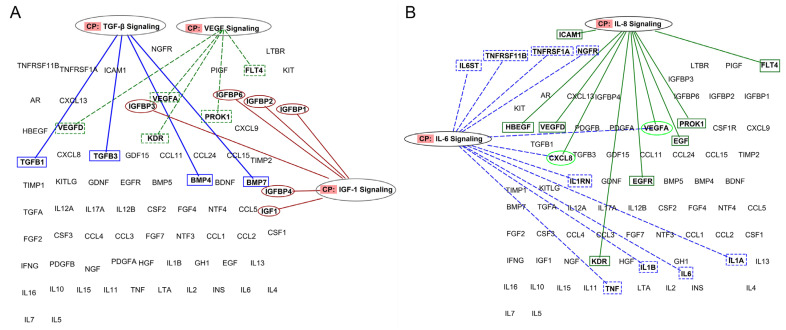
Representation of proteins involved in specific inflammatory and angiogenic pathways and their connections. (**A**) PRP actions should not be attributed to a single growth factor but to the interplay in PRP signaling networks (**B**) IL-8 and IL-6 signaling networks within plasma formulations (figures were created using Ingenuity Pathway Analysis (IPA), QIAGEN).

Bioinformatic analyses reveal that growth factor signaling, e.g., TGF-β, VEGF, and IGF-1, depends on protein networks rather than on the individual GFs. Figure 3A,B illustrates some of these relationships relevant to knee OA.

For example, the function of IGF-1 (known as an anabolic factor for cartilage) is controlled by several binding proteins (IGFBP-1, IGFBP-2, IGFBP-3, IGFBP-4, and IGFBP-6), which are very abundant in plasma (up to hundreds of nanograms). These proteins can hinder IGF-1 binding to its target receptor located in the cell membrane. TGF-β signaling is influenced by both TGF-β1 and TGF-β3 isoforms and by other members of TGF superfamily of proteins, i.e., BMP-4 and BMP-7. These proteins activate the SMAD family of transcription factors and can influence angiogenesis and osteogenesis. Similarly, both VEGF-A and VEGF-D proteins are relevant to angiogenesis, but their binding to membrane receptors can be hampered by VEGFR3 (FLT4) and VEGFR2 (KDR), which function as decoy receptor; thus, they function as negative regulators of VEGF actions. Besides, EG-VEGF (PROK1) is abundant in PRPs and can induce proliferation, migration, and fenestration in capillary endothelial cells.

Moreover, as shown in Figure 3B, IL8 signaling is influenced by the molecules involved in VEGF signaling (VEGF-A and -D, KDR, PROK1, and FLT4) and also interacts with IL-6 signaling. Both IL-6 and IL-8 are pleiotropic proteins that participate in inflammation and tissue homeostasis.

**Figure 4:**
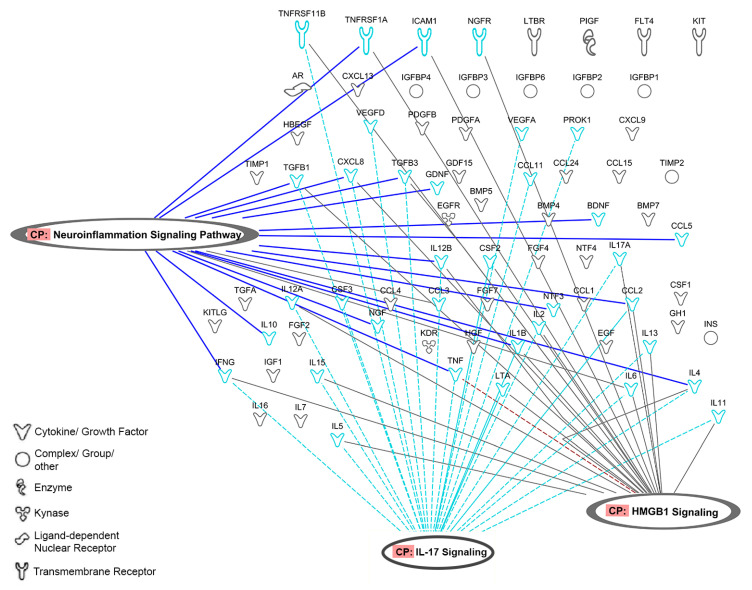
Representation of proteins involved in neuroinflammation signaling pathways and their connections with IL-17 and HMGB1 signaling (figure was created using Ingenuity Pathway Analysis (IPA), QIAGEN).

As shown in Figure 4, 20 cytokines from platelet secretome affect neuroinflammation pathways and share signaling cytokines with IL17 and HMGB1 inflammatory pathways (Table S2).

**Figure 5:**
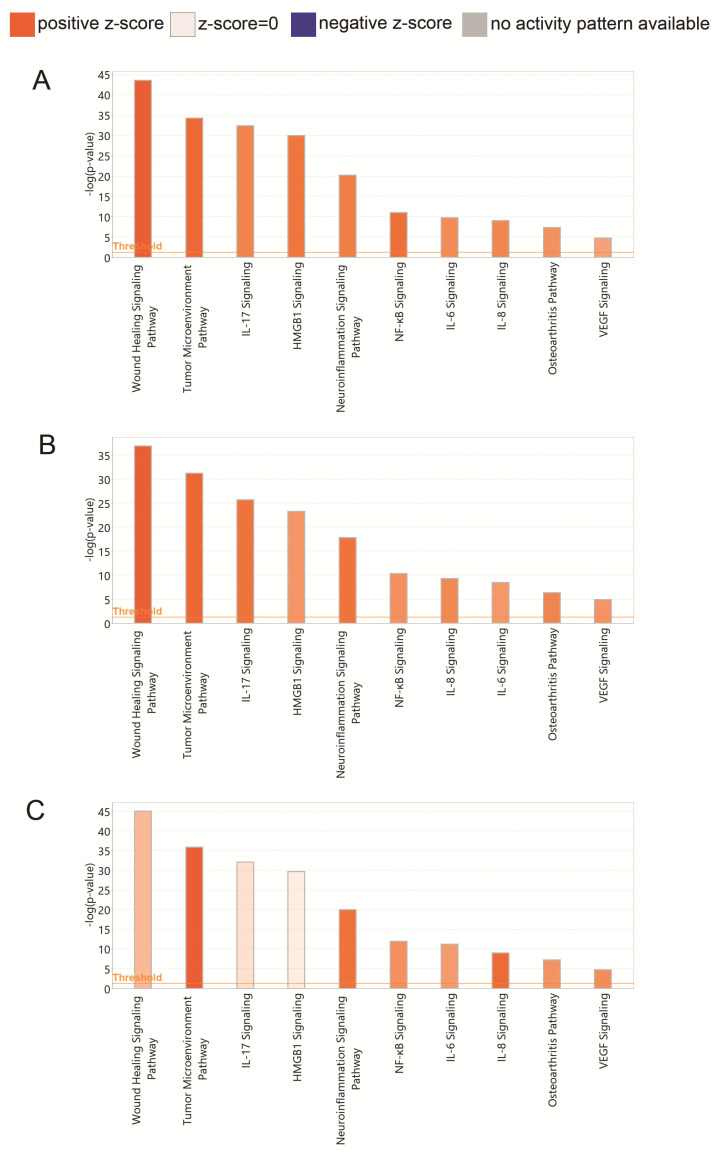
Functional pathways represented in the data: (**A**) predictions of canonical pathways activation for protein fold changes obtained with biobank plasma (PRP vs. PPP). (**B**) Same for in-house prepared plasmas. (**C**) Same for in-house prepared supernatants (collected 1 h after CaCl_2_ activation) *p*-values < 0.05 indicate non-random association between our experimental datasets and functions/pathways. (Fold changes for each preparation procedure are shown in Table S1). The colors of the bars represent IPA’s *z*-score, which predicts pathway activation or inhibition.

According to the IPA regulation *z*-score algorithm, wound healing mechanisms are more active in PRPs (*z*-scores 4.596 and 3.780 for biobank and in-house, respectively) than in the supernatant harvested one hour after plasma clotting (*z*-score 1.219). As a note of caution, all preparations activate the tumor microenvironment, and thus, the use of PRPs should be precluded in oncologic patients. According to bioinformatic predictions, the supernatant formulation has weaker influence in the activation of inflammatory pathways, including HMGB1 and IL-17 pathways (Figure 5).

## 4. Discussion

Intraarticular PRP for knee osteoarthritis reduces pain and improves function in some patients [10]. However, the lack of mechanistic insights hinders treatment optimization and selection of responders. Analyses of PRP proteins help to upgrade mechanistic research hypothesis in order to improve our understanding on the role of platelet secretome contextualized in the knee OA scenario. With this objective, we obtained PRP and PPP from the local blood bank, pooled samples from six donors before performing multiplexing protein arrays, and normalized PRP results to fold change in relation to PPP. In doing so, we analyzed the molecular implications of platelet secretome, which are believed to be the core elements in PRP actions. In fact, the main concept behind PRP treatment is to increase the concentration of platelets (and their secretory molecules) in the target tissue/organ. 

We focused on PRP (without leukocytes)(without stressing differences between formulations), as it is the most common formulation used in clinics [16], and current meta-analyses results refer to these PRP variants [20,21]. In parallel, we performed the same analyses for our in-house plasma preparations and found some differences in cluster analyses but similar results in terms of represented pathways. The former could be attributed to differences in the donor population or/and in the preparation procedure.

We did not conduct the whole proteomic analyses of plasma but concentrated on candidate protein arrays involved in PRP signaling in the context of OA. Nevertheless, our results are in accordance with most recent whole proteomic analyses [22] that revealed that the major regulatory proteins and pathways represented in PRP are related to inflammation. Indeed, bioinformatic predictions derived from this study confirm that PRP molecules influence inflammatory mechanisms and immune cell interactions. These results contravene current thinking and descriptions of PRPs focused above all on the growth factors [23,24] and their anabolic effects in chondrocytes as the basis of PRPs’ mechanism of action. Although the proliferative and anabolic actions of PRPs have been shown in vitro [25], it seems improbable that chondrocytes embedded within the deteriorated cartilage could proliferate and synthesize ECM. 

At present, the OA research scope has shifted from the cartilage-centric catabolic approach to the understanding of the joint as a whole organ made of several types of tissues and infiltrated by immune cells [26]. Still, OA attributes according to IPA annotations software represent only chondrocyte involvement in accordance with the historical concept, which considered OA as a “wear-and-tear disease” initiated by loss of cartilage integrity, which deteriorates progressively, exacerbating the disease during ageing [27]. 

In the last decade, OA has been recognized as a whole-organ disorder affecting various joint tissues in different degrees depending on the disease stage or the specific patient phenotype and endotype [28]; novel insights are anticipated using high-throughput OMIC technologies [29]. Moreover, OA is not a single entity, and current research focuses on personalized OA management according to OA subtypes and main drivers of disease progression [30].

PRP therapy is inevitably linked to advances in OA. Major changes have characterized OA research in the last decades. Mainly, OA has shifted from being a non-inflammatory disease because of the lack of inflammatory cell infiltration in the synovium and absence of typical systemic inflammatory manifestations, which characterized autoimmune arthritis [31] to a low-grade local inflammatory disease [32]. 

Unequivocal description of inflammatory changes is lacking; OA is not considered an autoimmune disease; yet, infiltration of CD4+ T cells has been shown in early stages [33]. According to big data analyses, there are differences between the immune cells’ subsets that infiltrate the OA joint tissues compared to controls [26]. For example, there are less M2 macrophages and a different pattern of activation of dendritic cells (higher number of activated dendritic cells, higher numbers of must resting cells, and differences in T-cells CD4 memory activated and resting) in the OA knee tissues than in controls. Targeting macrophage polarization is a current topic of research. At the molecular level, OA synovial fluid contains inflammatory molecules, including cytokines (i.e., TNF-α, IL-1β, IL-6), chemokines, adipokines, prostaglandins, and leukotrienes [34]. The activities of local and infiltrated macrophages have been associated to ECM debris phagocytosis and innate immune activation, both associated with disease progression [35]. During the healing process, macrophages polarize from M1 (inflammatory) to M2 (reparative) by passing through a range of intermediary molecular phenotypes. In failed healing circumstances, they do not reach M2, and as a result, they perpetuate inflammation and hinder homeostasis. Current research emphasizes the role of joint macrophages in the maintenance of homeostasis [36]. Molecular panel of inflammatory proteins has shown clinical validity to describe rapidly progressing OA phenotypes [37,38].

In this context, PRP molecules can influence the polarization of macrophages [39,40], producing different macrophage states and setting the basis for novel formulations specifically targeted to macrophage polarization. The present bioinformatic analysis predicts PRP intervention in macrophage biology and a role in modulating their crosstalk with other immune cells. However, protein arrays in young versus old PRP reveal differences with functional implications in chondrocytes and in macrophage polarization [41]. In fact, ageing involves cell senescence and immune cell dysfunction known as inflamageing [42]. Accordingly, in the older patients, autologous PRP is not pertinent, and allogeneic alternatives warrant investigation [43].

According to the opportunities that provide the above-described pool of proteins that could modulate inflammation in knee OA, one therapeutic approach is to modify the crosstalk between innate immune cells that infiltrate the joint and, in doing so, alter the vicious inflammatory-catabolic loop by modifying the molecular composition of the synovial fluid [44]. Because of the accessibility of synovial fluid through intraarticular needle, it is possible to evacuate inflammatory SF and substitute it by a pool of plasma cytokines and growth factors, a subset of which we reveal in this study.

Interestingly, predictions showed that PRP molecules modulate IL-17 signaling. Recent data support a role for IL-17A in OA joints [45] and set the grounds to investigate this specific inflammatory phenotype. IL-17 in increased in obese and associated to obesity-related inflammatory comorbidities [46]. Moreover, NF-kB pathway is modulated by PRP [47], and IL-17A significantly activates p38 and p65 NF-κB in synovial cells and chondrocytes. Activation of NK-kB leads to the release of catabolic enzymes (matrix metalloproteinase (MMP)-1,-3,-9, and -13), cytokines (tumor necrosis factor (TNF)-α, interleukin (IL)-1β, and IL-6), and C-C chemokines, i.e., CCL-2,-5,-7,-8, cathepsins, and complement cascade, factors described as essential in OA pathogenesis [48,49]. Although IL-17 concentration is very low in PRP, 20 additional components of the analyzed pool participate in the regulation of IL-17.

HMGB1 signaling is also modulated by PRP. HMGB1 is an alarmin present in the extracellular environment after chondrocyte or macrophage pyroptosis (death). Actually, this protein is a target in the development of miRNA therapies for OA [50], as early protein blockade can decrease cartilage deterioration [51]. Current predictions revealed that PRP influences HMGB1 signaling. On the other hand, pyroptosis of synovial fibroblasts and macrophages increase intraarticular levels of HMGB1 and have been associated with OA severity [52]. A priori, platelets could exacerbate inflammation, as they are a relevant source of HMGB1. However, PRP from HMGB1 KO mice cannot enhance tendon healing in contrast to normal PRP [53]. Notwithstanding with OA studies, the presence of HMGB1 decreases inflammation in tendinopathy and is necessary for tendon healing, an effect attributed to the chemotactic actions of HMGB1 in stem cells. Future studies are necessary to analyze the implications of PRP in HMGB1 regulation in OA.

Neuroinflammation signaling pathways play a crucial role in pain transmission. Pain is cardinal in OA, and clinical data indicate that PRPs downregulate pain not only in OA but also in other clinical applications [54]. The involvement of 20 PRP molecules in the active crosstalk between nociceptor neurons and immune system can help to understand the mechanisms by which PRPs can modulate pain [55], which is paramount from a clinical standpoint. Interestingly, according to these analyses, neuroinflammation pathways are represented in the experimental data sets: biobank, in house, and supernatant.

As a note of caution, PRP treatments should be avoided in oncologic patients, as platelet secretome can interact with the tumor microenvironment; 75 proteins from the pool we examined can participate in tumor progression. Interestingly, wound healing is represented in biobank and in-house data sets but not in the supernatants.

PRPs composition is very complex, as they contain very abundant plasma proteins, such as globulins, albumins, and many coagulation proteins, and our understanding of how they interfere in ligand-receptor interactions is very limited. Interpretation of the current results is limited because we have assessed only a subset of PRP proteins. In fact, this is an exploratory study, and instead of analyzing the whole proteomic content, we focused on specific signaling proteins, which compromises statistical findings.

Bioinformatic predictions help to establish novel hypothesis and future research directions considering molecular networks rather than single molecules. In the coming years, integration of PRP complexity into OA progression mechanisms will help to optimize PRPs in order to bridge the gap between palliative treatments and surgery tailoring PRP composition to the different inflammatory phenotypes. Only in doing so could PRPs become part of the recommendations and therapeutic guidelines, at least for specific patient phenotypes. We have great challenges and opportunities ahead, and with good research, we will meet them and optimize treatments; then, we will contribute to facilitating healthy retirements while fostering the 2021–2030 Decade of Healthy Aging.

## 5. Conclusions

Overall, our study provides a new way of thinking about PRP therapies, revealing that their predicted actions are related with the regulation of inflammatory processes and provide grounds for hypothesis formulation involving PRP actions in different inflammatory OA phenotypes and predict cytokines involvement in various inflammatory pathways and pain mechanisms. It will take time and research efforts to identify the confluence between PRP components and OA progression mechanisms, and complex OMIC tools will be needed.

## Figures and Tables

**Table 1:** Cell counts in the biobank blood samples.

Cells	Whole Blood	PPP Biobank	PRP Biobank
Platelets (×10^3^/µL)	220 ± 0.42	24 ± 13	1972 ± 325 (×8.97)
Leukocytes (×10^3^/µL)	5.2 ± 0.87	n.d.	3.91 ± 5.02 (0.56–11.39) (lymphocytes)
Erythrocytes (×10^6^/µL)	4.17 ± 0.48	n.d.	n.d.

n.d., non-detected.

**Table 2:** Cell counts in the in-house blood samples.

Cells	Whole Blood	PPP in-House	PRP in-House
Platelets (×10^3^/µL)	226 ± 41	15 ±	460 ± 103 (×2.03)
Leukocytes (×10^3^/µL)	4.9 ± 0.74	n.d.	0.06 ± 0.07 (lymphocytes)
Erythrocytes (×10^6^/µL)	4.38 ± 0.62	n.d.	n.d.

PRP, platelet rich plasma, PPP, platelet poor plasma. Biobank PRP and PPP: Selected Signaling Proteins Concentrations from Individual Donors.

**Table 3:** Molecular characterization (ELISA) of the individual components of the pools.

	Platelet-Rich Plasma (PRP)	Platelet-Poor Plasma (PPP)	*p*-Value
Total protein	243.78	225.16	
(BCA) mg/mL	264.91	251.96	
	193.61	246.44	
	234.75	219.07	
	233.07	223.61	
	262.4	240.98	
Mean (SD)	238.83 ± 25.72	234.50 ± 13.88	
Interdonor %CV	10.77%	5.92%	*p* = 1.000
PDGF ng/mL	23.77	5.5	
	24.13	5.59	
	38.31	5.79	
	17.12	5.86	
	29.7	6.32	
	21.75	5.33	
Mean (SD)	25.78 ± 7.36	5.73 ± 0.35	*p* = 0.002
Interdonor %CV	28.54%	6.10%	
VEGF pg/mL	277.19	94.17	
	639.08	145.42	
	901.23	215.21	
	725.91	273.50	
	751.44	223.59	
	604.32	170.69	
Mean (SD)	649.85 ± 209.91	187.10 ± 63.60	*p* = 0.002
Interdonor %CV	32.30%	33.99%	
MCP1 pg/mL	429.25	384.70	
	580.25	322.13	
	709.19	541.16	
	632.09	497.11	
	428.35	357.47	
	397.30	369.82	
Mean (SD)	529.38 ± 128.97	412.07 ± 86.62	*p* = 0.065
Interdonor %CV	24.36%	21.02%	
RANTES pg/mL	1080.25	71.66	
	1227.34	116.38	
	1462.00	90.40	
	1001.23	90.30	
	849.46	90.75	
	1183.94	110.01	
Mean (SD)	1133.92 ± 209.89	94.94 ± 16.05	*p* = 0.002
Interdonor %CV	18.51%	16.90%	

PRP, platelet rich plasma, PPP, platelet poor plasma, MCP-1, monocyte chemoattractant protein-1; VEGF, vascular endothelial growth factor; RANTES, regulated upon activation, normally T-expressed, and presumably secreted; PDGF-BB, platelet derived growth factor.

## Data Availability

The data presented in this study are available on request from the corresponding author.

## Supplementary Materials

The following supporting information can be downloaded at: https://www.mdpi.com/article/10.3390/jcm11030473/s1, Table S1: Experimental folds for the biobank, in-house, and supernatant formulations; Table S2: Biological attributes per protocol associated with experimental datasets.
