# Supplementary material for: Unraveling the Signaling Secretome of Platelet-Rich Plasma: Towards a Better Understanding of Its Therapeutic Potential in Knee Osteoarthritis
Source: J Clin Med. 2022 Jan 18;11(3):473. doi: 10.3390/jcm11030473 (PMC8836812; doi:10.3390/jcm11030473)
Supplement: Supplementary file 1 [file jcm-11-00473-s001.zip › jcm-1504434-supplementary.pdf]

Table S1. Experimental folds for the biobank, in-house, and supernatant formulations.

PRP Biobank-fold change	PRP in house fold change	SN Fold Change	ID	Symbol	Entrez Gene Name	Location	Type(s)
3.319	2.181	2.713	AR	AR	androgen receptor	Nucleus	ligand-dependent nuclear receptor
95.994	130.865	38.967	BDNF	BDNF	brain derived neurotrophic factor	Extracellular Space	growth factor
5.314	−2.001	2.492	BMP4	BMP4	bone morphogenetic protein 4	Extracellular Space	growth factor
3.051	2.721	2.925	BMP5	BMP5	bone morphogenetic protein 5	Extracellular Space	growth factor
3.445	1.874	2.199	BMP7	BMP7	bone morphogenetic protein 7	Extracellular Space	growth factor
−1.343	1.064	1.333	CCL1	CCL1	C-C motif chemokine ligand 1	Extracellular Space	cytokine
1.532	1.778	3.773	CCL11	CCL11	C-C motif chemokine ligand 11	Extracellular Space	cytokine
2.108	2.296	1.917	CCL15	CCL15	C-C motif chemokine ligand 15	Extracellular Space	cytokine
2.817	9.210	2.058	CCL2	CCL2	C-C motif chemokine ligand 2	Extracellular Space	cytokine
1.683	1.432	2.995	CCL24	CCL24	C-C motif chemokine ligand 24	Extracellular Space	cytokine
−1.261	5.625	−1.690	CCL3	CCL3	C-C motif chemokine ligand 3	Extracellular Space	cytokine
4.036	2.566	3.967	CCL4	CCL4	C-C motif chemokine ligand 4	Extracellular Space	cytokine
2.565	2.502	2.763	CCL5	CCL5	C-C motif chemokine ligand 5	Extracellular Space	cytokine
2.049	1.993	2.176	CSF1	CSF1	colony stimulating factor 1	Extracellular Space	cytokine
3.085	0.000	−1.372	CSF2	CSF2	colony stimulating factor 2	Extracellular Space	cytokine
−1.029	20.159	−1.404	CSF3	CSF3	colony stimulating factor 3	Extracellular Space	cytokine
1.297	−4.708	1.395	CXCL13	CXCL13	C-X-C motif chemokine ligand 13	Extracellular Space	cytokine
6.695	-1.813	1.735	IL8	CXCL8	C-X-C motif chemokine ligand 8	Extracellular Space	cytokine
−1.391	1.078	3.065	CXCL9	CXCL9	C-X-C motif chemokine ligand 9	Extracellular Space	cytokine
20.452	−22.360	−1.602	EGF	EGF	epidermal growth factor	Extracellular Space	growth factor
1.383	19.273	19.125	EGFR	EGFR	epidermal growth factor receptor	Plasma Membrane	kinase
3.807	1.473	1.249	FGF2	FGF2	fibroblast growth factor 2	Extracellular Space	growth factor
2.427	2.022	2.793	FGF4	FGF4	fibroblast growth factor 4	Extracellular Space	growth factor
2.306	2.060	2.394	FGF7	FGF7	fibroblast growth factor 7	Extracellular Space	growth factor
12.721	1.418	1.378	FLT4	FLT4	fms related receptor tyrosine kinase 4	Plasma Membrane	transmembrane receptor
1.489	4.958	8.506	GDF15	GDF15	growth differentiation factor 15	Extracellular Space	growth factor

3.560	2.360	3.001	GDNF	GDNF	glial cell derived neurotrophic factor	Extracellular Space	growth factor
2.361	1.908	2.638	GH1	GH1	growth hormone 1	Extracellular Space	growth factor
1.672	−1.235	2.563	HBEGF	HBEGF	heparin binding EGF like growth factor	Extracellular Space	growth factor
4.331	1.765	2.125	HGF	HGF	hepatocyte growth factor	Extracellular Space	growth factor
1.434	3.629	5.935	ICAM1	ICAM1	intercellular adhesion molecule 1	Plasma Membrane	transmembrane receptor
5.970	−1.106	2.050	IFNG	IFNG	interferon gamma	Extracellular Space	cytokine
7.063	14.949	−1.857	IGF1	IGF1	insulin like growth factor 1	Extracellular Space	growth factor
2.228	1.475	2.987	IGFBP1	IGFBP1	insulin like growth factor binding protein 1	Extracellular Space	other
2.099	1.882	4.023	IGFBP2	IGFBP2	insulin like growth factor binding protein 2	Extracellular Space	other
2.842	2.318	2.330	IGFBP3	IGFBP3	insulin like growth factor binding protein 3	Extracellular Space	other
1.788	2.056	2.444	IGFBP4	IGFBP4	insulin like growth factor binding protein 4	Extracellular Space	other
1.985	−1.141	1.080	IGFBP6	IGFBP6	insulin like growth factor binding protein 6	Extracellular Space	other
3.000	−1.077	1.374	IL10	IL10	interleukin 10	Extracellular Space	cytokine
5.258	11.638	1.052	IL11	IL11	interleukin 11	Extracellular Space	cytokine
3.721		-3.416	IL12A	IL12A	interleukin 12A	Extracellular Space	cytokine
-3.295		-1.967	IL12B	IL12B	interleukin 12B	Extracellular Space	cytokine
3.038	−1.528	−1.843	IL13	IL13	interleukin 13	Extracellular Space	cytokine
2.207	11.208	−1.002	IL15	IL15	interleukin 15	Extracellular Space	cytokine
1.022	1.266	3.688	IL16	IL16	interleukin 16	Extracellular Space	cytokine
1.702	−2.043	2.177	IL17	IL17A	interleukin 17A	Extracellular Space	cytokine
2.030		−2.784	IL1B	IL1B	interleukin 1 beta	Extracellular Space	cytokine
1.252		0.000	IL2	IL2	interleukin 2	Extracellular Space	cytokine
−1.057	0.000	2.927	IL4	IL4	interleukin 4	Extracellular Space	cytokine
2.076	0.000	−2.721	IL5	IL5	interleukin 5	Extracellular Space	cytokine
6.213	2.192	−2.086	IL6	IL6	interleukin 6	Extracellular Space	cytokine
3.282	0.000	1.276	IL7	IL7	interleukin 7	Extracellular Space	cytokine
3.602	4.966	−1.559	INS	INS	insulin	Extracellular Space	other
3.617	7.940	2.366	KDR	KDR	kinase insert domain receptor	Plasma Membrane	kinase

2.221	6.572	2.219	KIT	KIT	KIT proto-oncogene. receptor tyrosine kinase	Plasma Membrane	transmembrane receptor
2.312	1.787	3.183	KITLG	KITLG	KIT ligand	Extracellular Space	growth factor
3.050	2.185	2.161	LTA	LTA	lymphotoxin alpha	Extracellular Space	cytokine
1.193	1.514	2.002	LTBR	LTBR	lymphotoxin beta receptor	Plasma Membrane	transmembrane receptor
4.401	1.647	2.138	NGF	NGF	nerve growth factor	Extracellular Space	growth factor
3.663	11.200	−1.811	NGFR	NGFR	nerve growth factor receptor	Plasma Membrane	transmembrane receptor
4.470	2.258	1.997	NTF3	NTF3	neurotrophin 3	Extracellular Space	growth factor
2.303	1.393	2.262	NTF4	NTF4	neurotrophin 4	Extracellular Space	growth factor
5.800	1.352	2.384	PDGFA	PDGFA	platelet derived growth factor subunit A	Extracellular Space	growth factor
4.320	1.957	4.092	PDGFB	PDGFB	platelet derived growth factor subunit B	Extracellular Space	growth factor
1.570	2.620	2.965	PIGF	PIGF	phosphatidylinositol glycan anchor biosynthesis class F	Cytoplasm	enzyme
2.766	4.628	4.718	PROK1	PROK1	prokineticin 1	Extracellular Space	growth factor
1.084	7.804	7.895	TGFA	TGFA	transforming growth factor alpha	Extracellular Space	growth factor
2.407	1.257	1.418	TGFB1	TGFB1	transforming growth factor beta 1	Extracellular Space	growth factor
2.254	4.249	2.298	TGFB3	TGFB3	transforming growth factor beta 3	Extracellular Space	growth factor
1.991	2.632	−1.318	TIMP1	TIMP1	TIMP metallopeptidase inhibitor 1	Extracellular Space	cytokine
2.604	2.909	2.318	TIMP2	TIMP2	TIMP metallopeptidase inhibitor 2	Extracellular Space	other
2.873	−1.059	1.731	TNF	TNF	tumor necrosis factor	Extracellular Space	cytokine
2.345	2.014	2.076	TNFRSF 11B	TNFRSF 11B	TNF receptor superfamily member 11b	Plasma Membrane	transmembrane receptor
1.287	3.080	3.002	TNFRSF 1A	TNFRSF 1A	TNF receptor superfamily member 1A	Plasma Membrane	transmembrane receptor
3.476	4.946	−1.902	VEGFA	VEGFA	vascular endothelial growth factor A	Extracellular Space	growth factor
4.428	−2.897	2.918	VEGFD	VEGFD	vascular endothelial growth factor D	Extracellular Space	growth factor

Table S2. Biological attributes per protocol associated with experimental datasets. Molecule ratios denote molecules present in our dataset/those known to be associated with the annotation. -log (*p*-value) is *p*-value of overlap, which measures the significance /enrichment of a biological attribute.

**Activated functions/pathways.**	**Enrichment score per protocol (molecule ratio) –log(*p*-value)**		
	**Biobank**	**In-house**	**Supernatant**
Wound healing	32/ 256<br>43.57	28/256<br>37.128	34/256<br>47.201
Tumour microenvironment	25/188<br>34.29	23/188<br>31.208	26/188<br>35.839
IL-17 signalling	24/189<br>32.36	20/189<br>25.714	24/189<br>32.044
HMGB1 signalling	22/167<br>29.951	18/167<br>23.44	22/167<br>29.66
Neuroinflammation	20/330<br>20.272	18/330<br>17.854	20/330<br>20.023
NF-kB signalling	16/570<br>11.015	15/570<br>10.296	17/570<br>11.924
IL-8	10/215<br>9.07	10/215<br>9.307	10/215<br>8.955
IL-6	9/128<br>9.793	8/128<br>8.535	10/128<br>11.18
OA pathway	9/241<br>7.377	8/241<br>6.447	9/241<br>7.277
